# Sociocultural and Demographic Factors Predict Readmissions for General Surgery Patients

**DOI:** 10.1007/s00268-023-07177-0

**Published:** 2023-09-29

**Authors:** Joshua G. Kovoor, Stephen Bacchi, Aashray K. Gupta, Silas D. Nann, Brandon Stretton, Esther H. L. Chong, Joseph N. Hewitt, Ameya Bhanushali, Kayla Nathin, Nidhi Aujayeb, Amy Lu, Christopher D. Ovenden, Athul John, Jessica L. Reid, Samuel Gluck, Danny Liew, Benjamin A. Reddi, Thomas J. Hugh, Christopher Dobbins, Robert T. Padbury, Peter J. Hewett, Markus I. Trochsler, Guy J. Maddern

**Affiliations:** 1 Department of Surgery, The University of Adelaide, The Queen Elizabeth Hospital, 28 Woodville Road, Woodville South, SA Australia; 2 Royal Australasian College of Surgeons, Adelaide, SA Australia; 3 Port Augusta Hospital, Port Augusta, SA Australia; 4 Flinders Medical Centre, Flinders University, Adelaide, SA Australia; 5 Royal Adelaide Hospital, Adelaide, SA Australia; 6 University of Adelaide, Adelaide, SA Australia; 7 Gold Coast University Hospital, Gold Coast, QLD Australia; 8 University of Sydney, Sydney, NSW Australia; 9 Royal North Shore Hospital, Sydney, NSW Australia; 10 Flinders Medical Centre, Adelaide, SA Australia

## Abstract

**Introduction:**

Readmission is a poor outcome for both patients and healthcare systems. The association of certain sociocultural and demographic characteristics with likelihood of readmission is uncertain in general surgical patients.

**Method:**

A multi-centre retrospective cohort study of consecutive unique individuals who survived to discharge during general surgical admissions was conducted. Sociocultural and demographic variables were evaluated alongside clinical parameters (considered both as raw values and their proportion of change in the 1–2 days prior to admission) for their association with 7 and 30 days readmission using logistic regression.

**Results:**

There were 12,701 individuals included, with 304 (2.4%) individuals readmitted within 7 days, and 921 (7.3%) readmitted within 30 days. When incorporating absolute values of clinical parameters in the model, age was the only variable significantly associated with 7-day readmission, and primary language and presence of religion were the only variables significantly associated with 30-day readmission. When incorporating change in clinical parameters between the 1–2 days prior to discharge, primary language and religion were predictive of 30-day readmission. When controlling for changes in clinical parameters, only higher comorbidity burden (represented by higher Charlson comorbidity index score) was associated with increased likelihood of 30-day readmission.

**Conclusions:**

Sociocultural and demographic patient factors such as primary language, presence of religion, age, and comorbidity burden predict the likelihood of 7 and 30-day hospital readmission after general surgery. These findings support early implementation a postoperative care model that integrates all biopsychosocial domains across multiple disciplines of healthcare.

**Supplementary Information:**

The online version contains supplementary material available at 10.1007/s00268-023-07177-0.

## Introduction

Readmission after hospital discharge is a poor outcome for patients and surgical systems. After general surgery, readmissions are known to be associated with postoperative complications [1]. Previous studies have used strategies such as nomograms integrating clinical characteristics to predict readmission with moderate success [2, 3]. However, patient demographic factors, which can represent aspects of sociocultural support structures, can often be underappreciated when evaluating readmissions and assessing fitness for hospital discharge after general surgery. Accordingly, this study aimed to identify sociocultural and demographic factors that may predict 7-day and 30-day readmissions for general surgery patients.

## Methods

### Study setting and population

This study followed STROBE reporting guidelines [4]. A retrospective cohort study was conducted including consecutive elective and emergency adult patients admitted under general surgery services (colorectal, upper gastrointestinal, surgical trauma, hepatobiliary and acute surgical units) at two tertiary hospitals in South Australia over a two-year period commencing April 2020. This time period was chosen to ensure the highest possible reliability of data within the institutional electronic medical record used. Within this healthcare system, all patients have access to the same healthcare resources regardless of income or poverty level. Discharge planning at both participating institutions had access to the same resources and services as they were within the same healthcare network, however specific protocols regarding postoperative discharge planning varied between each individual treating team. Discharge planning within the surgical units of the participating institutions usually considered the patient’s social network, need for home care and supports, planned outpatient follow-up visits, and need for post-discharge monitoring. There was no specialized team that specifically took care of post-discharge follow-up and patient needs for the included surgical units, however for cases of complex discharge planning there was specialised staff members available that could be consulted to provide assistance. Initial presentations resulting in in-hospital mortality were excluded. Readmission was defined as requiring repeat admission at one of the two participating institutions, and two readmission time cut-offs were employed of 7 and 30-days. Within analysis, distinction was made between 7 and 30-day timepoints for readmission rates for additional clinical relevance and clarity, using the logic that readmissions within 7 days are more likely to be directly related to the surgery itself, while those extending out to 30 days have a greater likelihood that other factors, such as sociocultural or community variables and adjuvant therapy, play a contributory role. Hospital readmission at the participating institutions is reliably automatically captured within the institutional electronic medical record, however readmissions to hospitals other than the participating institutions was not reliably captured and outside the scope of the present study. Within the institutional electronic medical record that was utilised, mortality data are regularly collected, matched, and cross-checked with the South Australian Births, Deaths and Marriages registry at monthly intervals [5]. Accordingly, deaths that occur after discharge without readmission in the first 30 days are reliably captured within the electronic medical record, regardless of whether they occur in a hospital or in the South Australian community. Ethical approval was granted by the Central Adelaide Local Health Network Human Research Ethics Committee (reference number: 16409), with a waiver of individual consent.

### Data collection

Data were prospectively entered within the electronic medical record by clinical staff throughout each patient admission, and then were retrospectively collected for the present study. The presence or absence of demographic characteristics, such as religion, was extracted as entered within the institutional electronic medical record. These data were entered by administrative staff when each patient was admitted to hospital, based on what was communicated to them by the patient. Although the present study aimed to identify patient sociocultural and demographic factors that predict general surgery readmissions, various clinical parameters that are commonly collected in general surgery admissions were also collected and included within analyses to provide clinical relativity of subsequent significant findings. For socioeconomic status calculation, domestic region is integrated within the Socio-Economic Indexes for Areas (SEIFA) product from the Australian Bureau of Statistics to rank postcodes in terms of socioeconomic status as a percentile relative to that state (South Australia in this instance) [6]. SEIFA is based on the five-yearly Australian Census of Population and Housing, and integrates four indexes: The Index of Relative Socio-economic Disadvantage (IRSD); The Index of Relative Socio-economic Advantage and Disadvantage (IRSAD); The Index of Education and Occupation (IEO); The Index of Economic Resources (IER) [6]. Details of this methodology are described on the Australian Bureau of Statistics website [6].

### Statistical analysis

Descriptive statistical analyses were used to characterise the cohort. As mentioned above, although the present study aimed to identify sociocultural and demographic factors that predict general surgery readmissions, various clinical parameters that are commonly collected in general surgery admissions were also collected and included within analyses to provide clinical relativity of subsequent significant findings. To investigate the effect of temporal proximity to hospital discharge, within the statistical models, clinical parameters were incorporated in two time-intervals based on their time of collection by treating staff: within 24 h (1 day) of discharge, and 24–48 h (1–2 days) prior to discharge. Change between 1–2 days prior to discharge was calculated using subtraction. When more than one clinical parameter was collected within the two time periods, the maximum result was included in analysis. Median imputation was used to replace missing input data. Multivariable logistic regression was used to evaluate for factors independently associated with readmission within 7 or 30-days. Explanatory variables for the multivariable logistic regression analyses included sociocultural and demographic variables (age, gender, Charlson [7] comorbidity index, socioeconomic percentile), and clinical parameters commonly collected during general surgery admissions (respiratory rate, oxygen saturations, supplemental oxygen requirement, heart rate, systolic blood pressure, temperature, sedation score, bowel openings per day, pain score (/5), haemoglobin, white cell count, neutrophil count, lymphocyte count, platelets, sodium, potassium, creatinine, urea, magnesium, ALP, AST, ALT, GGT, bilirubin, albumin, LDH, calcium, glucose, phosphate, APTT, PT, INR, and CRP). The inclusion of the clinical parameters was to provide clinical relativity of subsequent significant findings Analysis was performed using R and Python (version 1.1.456).

## Results

12,846 individuals were admitted during the study period, however 145 experienced in-hospital mortality. Accordingly, 12,701 patients were included for analysis. Of these, mean age was 54.6 (SD 20.4) and 6099 (48.0%) were female sex. The number of patients with a readmission within 7 days was 304 (2.4%), and within 30 days was 921 (7.3%). The complete cohort characteristics, delineated based on presence of 30-day readmission, are presented in Table 1. The clinical variables that were significantly associated with 7-day and 30-day readmission within logistic regression analyses, are presented in the supplementary appendix.

**Table 1:** Cohort characteristics in the 24 h prior to discharge

Variable	Individuals not readmitted within 30-days (*n* = 11,780)	Individuals readmitted within 30-days (*n* = 921)	*P* value
Age; mean (SD)	54.6 (20.5)	55.5 (19.7)	0.18
Female sex; number (%)	5646 (47.9)	453 (49.2)	0.48
Socioeconomic percentile; mean (SD)	59.0 (28.5)	59.6 (27.7)	0.57
Non-English primary language; number (%)	1036 (8.8)	113 (12.2)	<0.001
No specified religion; number (%)	3687 (31.3)	340 (36.9)	<0.001
Respiratory rate; mean (SD)	17.7 (2.3)	17.7 (1.8)	0.86
Oxygen saturations; mean (SD)	98.5 (1.6)	98.5 (1.4)	0.21
Heart rate; mean (SD)	84.0 (12.9)	84.5 (13.0)	0.28
Systolic blood pressure; mean (SD)	137.9 (18.4)	137.5 (18.2)	0.55
Temperature; mean (SD)	37.0 (0.4)	37.1 (0.5)	0.10
Pain score (/5); mean (SD)	3.5 (2.9)	3.4 (2.9)	0.21
Haemoglobin; mean (SD)	123.0 (18.2)	122.3 (19.0)	0.38
White cell count; mean (SD)	8.7 (3.8)	8.7 (3.5)	0.80
Creatinine; mean (SD)	80.0 (53.7)	77.9 (37.9)	0.37
ALP; mean (SD)	118.6 (137.1)	154.9 (197.3)	<0.001
AST; mean (SD)	46.6 (70.2)	57.4 (87.6)	<0.01
ALT; mean (SD)	56.6 (96.9)	76.0 (124.2)	<0.001
GGT; mean (SD)	126.6 (224.5)	189.1 (316.8)	<0.001
Bilirubin; mean (SD)	13.6 (22.6)	20.7 (41.7)	<0.001
Albumin; mean (SD)	30.8 (4.9)	30.5 (4.8)	0.15
LDH; mean (SD)	228.7 (90.0)	234.0 (80.9)	0.21
Glucose; mean (SD)	6.9 (2.6)	6.9 (2.8)	0.67
C-reactive protein; mean (SD)	57.5 (59.0)	64.6 (65.5)	0.10

### 7-Day readmission

When incorporating absolute values of clinical parameters in the statistical model, age was the only sociocultural or demographic variable significantly associated with 7-day readmission. Increased age was associated with lower likelihood of readmission (OR 0.988, 95% CI 0.981 to 0.996, *P* = 0.002). When incorporating changes in clinical parameter values between 1 and 2 days prior to discharge in the statistical model, no sociocultural or demographic factors were significantly associated with 7-day readmission.

### 30-Day readmission

**Fig. 1:**
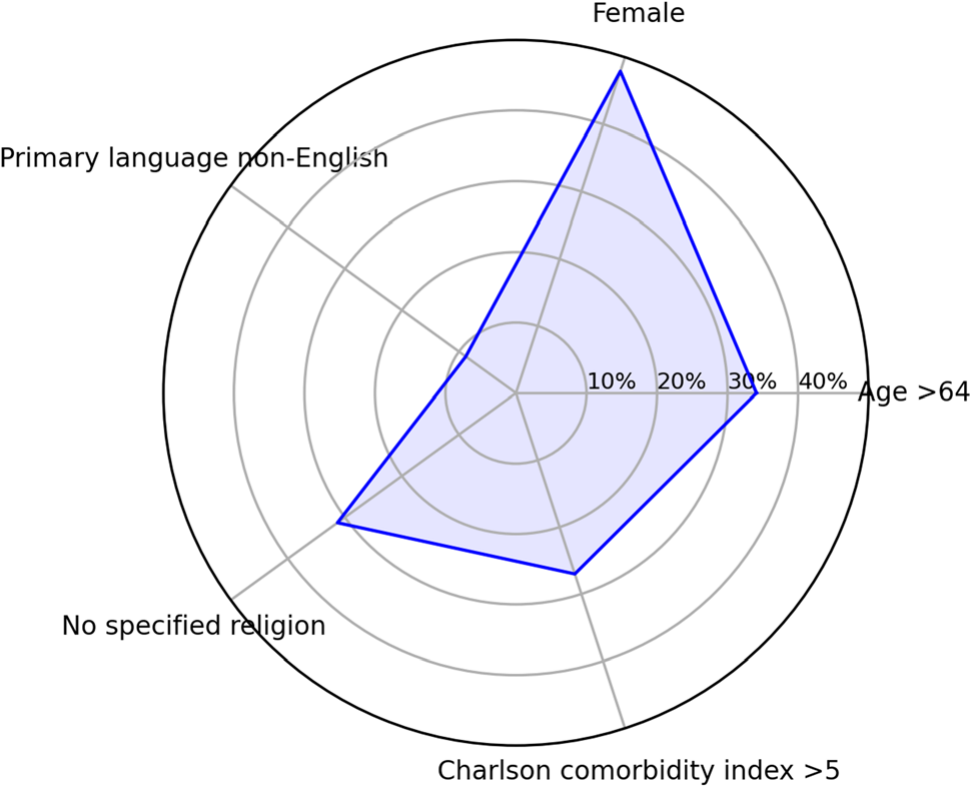
Sociocultural and demographic characteristics of patients not admitted within 30 days, as proportions

**Fig. 2:**
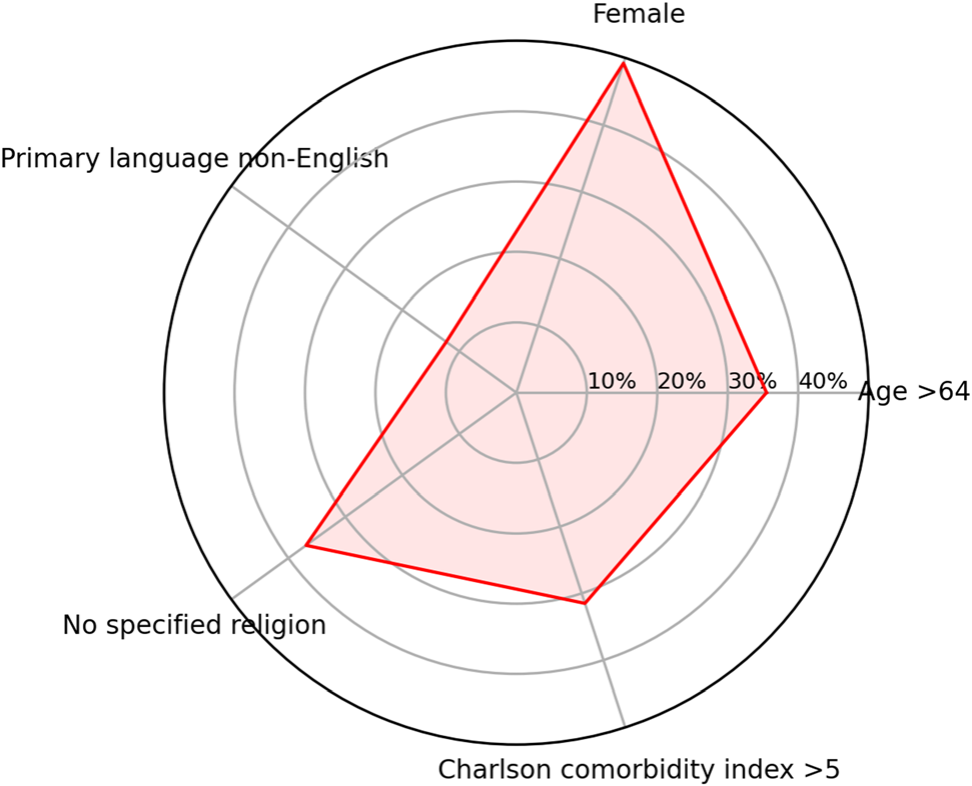
Sociocultural and demographic characteristics of patients readmitted within 30 days, as proportions

A graphical representation of the proportion of the 30-day readmitted patients relative to different sociocultural and demographic characteristics are presented in Figs. 1 and 2. When incorporating absolute values of clinical parameters into the statistical model, primary language and presence of religion were significantly associated with 30-day readmission. Having a primary language other than English (OR 1.3, 95% CI 1.046 to 1.615, *P* = 0.0018) and having no specified religion (OR 1.26, 95% CI 1.08 to 1.46, *P* = 0.003) were both associated with increased rates of 30-day readmission. When incorporating change in clinical parameters between 1 and 2 days prior to discharge in the statistical model, again primary language and religion were predictive of 30-day readmission. In particular, having a primary language other than English (OR 1.34, 95% CI 1.083 to 1.664, *P* = 0.007) and having no specified religion (OR 1.229, 95% CI 1.06 to 1.425, *P* = 0.006) were associated with an increased likelihood of 30-day readmission. When controlling for changes in clinical, only higher Charlson comorbidity index score was associated with increased likelihood of 30-day readmission (OR 1.029, 95% CI 1.003 to 1.055, *P* = 0.027).

## Discussion

This study identified that sociocultural and demographic factors including primary language, presence of religion, age, and comorbidity burden predict 7-day and 30-day general surgery readmissions. When interpreting the study findings, it must be noted that distinction was made between 7 and 30-day timepoints for readmission rates for additional clinical relevance and clarity, as readmissions within a week are more likely to be directly related to the surgery itself, while those extending out to a month have a greater likelihood that other factors, such as sociocultural or community variables and adjuvant therapy, play a contributory role. The overall 30-day readmission rates found in the present South Australian study are lower than those reported in some of the existing US literature [8], and it is important to note that this does not indicate any difference in quality of the respective systems of care, but merely sociocultural differences within the patient population and their milieu. These findings support the need to employ a holistic multi-disciplinary biopsychosocial model of care [9] when managing general surgical patients that considers evidence-based social determinants of health [10]. In particular, additional interventions to improve healthcare equity after hospital discharge for culturally and linguistically diverse patient population, such as language-diverse follow-up phone calls, should be investigated in future research. Consideration of strength of patient sociocultural and community support structures should be integrated within planning hospital discharge after general surgery.

This study adds novel data highlighting the importance of demographic and sociocultural factors for general surgical outcomes, to an existing literature that has primarily identified that postoperative complications are (intuitively) associated with increased rates of readmission after general surgery [1]. Findings of primary language and presence of religion being associated with readmission build on an evidence base that has identified that other social issues such as substance abuse and homelessness can contribute to readmission rates [11]. Language barrier has been relatively underexamined in several aspects of healthcare, although previous non-surgical studies have identified that it may predictor of readmission [12]. Increased age was associated with decreased readmission rates, which can be interpreted as counterintuitive, but may potentially represent increased rigour of care and post-discharge planning during the patient’s index admission. However, although this explanation is plausible it is also speculative, and future research investigating this association in more granular detail is required before system modification or subsequent evidence-based interventions can be made.

Although it may have been expected that patients with lower socioeconomic status may have experienced earlier readmissions, this was not found in the present study. Socioeconomic percentile was not associated with either 7 or 30-day readmission rates, and no significant difference in socioeconomic percentile was found between patients who experienced readmissions within 30-days versus those that did not. This suggests that sociocultural and community factors other than pure monetary wealth are likely playing a substantial role, such as level of social support provided by the patient’s close family and friend network. These findings are in contrast to other studies within the recent relevant surgical literature. In 2016, a prominent study by Glance et al. [13] found that differences in readmission rates after surgery between hospitals was due to differences in patient socioeconomic status, and not differences in hospital quality. Similarly, in 2020 Ghirimoldi et al. [14] found that patients residing in deprived communities of lower socioeconomic status were at a greater risk of readmission after colorectal surgery. However, in a large study investigating risk factors for readmission after emergency general surgery, Havens et al. [15] found that patient characteristics and behaviours other than socioeconomic status were notable risk factors, such as self-discharging against medical advice, having a greater comorbidity burden, and having public (as opposed to private) health insurance. Overall, the present study’s findings in the context of the relevant recent surgical evidence base suggest that the scope of investigation for non-clinical contributors to readmission after general surgery should be broadened within future research.

This study has limitations. Only English-speaking centres were included, and given the study findings, further research in centres speaking other languages is required. Type of surgery, which would impact a patient’s likelihood of readmission, was not accounted for in the present study. Patient ethnicity and gender present variables that were not investigated in this study, but are important considerations for future research. As institutionally-standardised coding was not automated within the electronic medical record from which the study’s data were derived, limitations can be found in details of the operations and postoperative admission not being accounted for within analyses for association with likelihood of readmission including type of surgery, open versus laparoscopic versus robotic approaches, complications, risk of infection, elective versus emergency admission, and major versus minor magnitude. As the data for the analyses within this study were dependent on the available values within the institutional electronic medical record from which the data were derived, laboratory values are over-represented, whereas surgical and demographic details are lacking. Specific procedural, postoperative course, and readmission aetiological breakdown were not possible to obtain from the electronic medical record, as manually coding for such a large sample size was seen as impractical given it would have required pearling of thousands of free-text operation notes, postoperative admission notes and data fields. Data regarding the location of patient discharge, such as directly to home versus other facilities, was not reliably entered within the institutional electronic medical record that was used for this study, and accordingly these data were not included as part of the associated analyses. Readmissions were examined within two centres in the same health network. While it is most likely that a patient would re-present to the same hospital to which they were recently admitted, it is likely that some readmissions to other centres [15], including private hospitals, were not captured. Accordingly, loss to follow-up due to readmissions elsewhere presents a limitation, particularly as patients from farther away distances tend to seek care in local facilities or providers before going back to index centers. Within the relevant literature, a prominent study by Tsai et al. described this care fragmentation in the postdischarge period, finding that 25% of 30-day readmissions after major surgery are to a hospital other than that where the index operation was performed, resulting in significantly increased risk of mortality even when travel distance is controlled for [16]. Similarly, a study by Turrentine et al. [17] found that each 10-min increase in travel time from patient residence to nearest hospital is associated with a 9% increase in likelihood of readmission after general surgery. An investigation regarding the association between lack of religion and socioeconomic status was outside the scope of the present study. Data comprising a direct comparison or evaluation between the health literacy of South Australia relative to that of the USA or other countries were not available. Further, within the associated populations (both South Australia, the USA, and other countries) there are a range of socioeconomic classes and levels of health literacy, making it challenging to conduct this direct juxtaposition. Future research should seek to conduct similar analyses using comprehensive national databases incorporating a prolonged timeframe and various patient populations detailed by coded information.

## Conclusions

Sociocultural and demographic patient factors such as primary language, presence of religion, age, and comorbidity burden predict the likelihood of 7 and 30-day hospital readmission after general surgery. These findings support early implementation a postoperative care model that integrates all biopsychosocial domains across multiple disciplines of healthcare. Additional interventions to improve healthcare equity after hospital discharge for culturally and linguistically diverse patient population, such as language-diverse follow-up phone calls, should be investigated in the future. Further research confirming causality within the associations found in the present study, followed by interventional studies trialling system strategies that take these findings into account in a manner that doesn’t result in healthcare inequity or disparity, should be encouraged across a diverse range of settings and patient populations.

### Supplementary Information

Below is the link to the electronic supplementary material.

Supplementary file1 (DOCX 15 kb)

## References

[1] Kassin MT, Owen RM, Perez SD (2012). Risk factors for 30-day hospital readmission among general surgery patients. J Am Coll Surg.

[2] Tevis SE, Weber SM, Kent KC (2015). Nomogram to predict postoperative readmission in patients who undergo general surgery. JAMA Surg.

[3] Muthuvel G, Tevis SE, Liepert AE (2014). A composite index for predicting readmission following emergency general surgery. J Trauma Acute Care Surg.

[4] Von Elm E, Altman DG, Egger M (2007). The strengthening the reporting of observational sudies in epidemiology (STROBE) statement: guidelines for reporting observational studies. Ann Intern Med.

[5] Government of South Australia Births, deaths and marriages, 2023

[6] Australian Bureau of Statistics 2033.0.55.001-Census of Population and Housing: Socio-Economic Indexes for Areas (SEIFA), Australia, 2016, 2018

[7] Charlson ME, Pompei P, Ales KL (1987). A new method of classifying prognostic comorbidity in longitudinal studies: development and validation. J Chronic Dis.

[8] Morris MS, Graham LA, Richman JS (2016). Postoperative 30-day readmission. Ann Surg.

[9] Borrell-Carrió F, Suchman AL, Epstein RM (2004). The biopsychosocial model 25 years later: principles, practice, and scientific inquiry. The Annals of Family Medicine.

[10] Marmot M, Wilkinson R, Social determinants of health (2005) Oup Oxford

[11] McIntyre LK, Arbabi S, Robinson EF (2016). Analysis of risk factors for patient readmission 30 days following discharge from general surgery. JAMA Surg.

[12] Squires A, Ma C, Miner S (2022). Assessing the influence of patient language preference on 30 day hospital readmission risk from home health care: a retrospective analysis. Int J Nurs Stud.

[13] Glance LG, Kellermann AL, Osler TM (2016). Impact of risk adjustment for socioeconomic status on risk-adjusted surgical readmission rates. Ann Surg.

[14] Ghirimoldi FM, Schmidt S, Simon RC (2021). Association of socioeconomic area deprivation index with hospital readmissions after colon and rectal surgery. J Gastrointest Surg.

[15] Havens JM, Olufajo OA, Cooper ZR (2016). Defining rates and risk factors for readmissions following emergency general surgery. JAMA Surg.

[16] Tsai TC, Orav EJ, Jha AK (2015). Care fragmentation in the postdischarge period: surgical readmissions, distance of travel, and postoperative mortality. JAMA Surg.

[17] Turrentine FE, Buckley PJ, Sohn M-W (2017). Travel time influences readmission risk: geospatial mapping of surgical readmissions. Am Surg.

