# Supplementary material for: Sociocultural and Demographic Factors Predict Readmissions for General Surgery Patients
Source: World J Surg. 2023 Sep 29;47(12):3124–30. doi: 10.1007/s00268-023-07177-0 (PMC10694098; doi:10.1007/s00268-023-07177-0)
Supplement: Supplementary file 1 — Supplementary file1 (DOCX 15 kb) [file 268_2023_7177_MOESM1_ESM.docx]

**SUPPLEMENTARY APPENDIX**

**Clinical variables that displayed significant association with 7-day and 30-day readmission within logistic regression analyses**

**Factors associated with 7-day readmission**

When examining absolute values (rather than change in values between the 24-hour and 24-to-48-hours periods), several parameters were associated with increased likelihood of readmission. A greater number of bowel movements in the preceding 24-hours was associated with increased likelihood of readmission (OR 0.767, 95%CI 0.64 to 0.921). A higher temperature (OR 1.335, 95%CI 1.015 to 1.756, P = 0.039) and greater sedation score (OR 1.22, 95%CI 1.008 to 1.477, P = 0.039) were similarly associated with higher likelihood of readmission. With respect to laboratory parameters, higher platelets were associated with a lower likelihood of readmission (OR 0.998, 95%CI 0.996 to 0.999, P = 0.017) and higher albumin was associated with a greater likelihood of readmission (OR 1.048, 95%CI 1.001 to 1.099, P = 0.048). With respect to changes in values between the 24-hour and 24-to-48-hours periods, a greater reduction in change in temperature (temperature going from higher to lower) was associated with a lower likelihood of readmission (OR 0.717, 95%CI 0.562 to 0.915, P = 0.008). Conversely, it could be said that an increase in temperature was associated with an increased likelihood of readmission. A greater reduction in bilirubin (bilirubin going from higher to lower) was associated with a lower likelihood of readmission (OR 0.991, 95%CI 0.982 to 0.999, P = 0.03). Similarly, a greater reduction in ALT (ALT going from higher to lower) was associated with a lower likelihood of readmission (OR 0.998, 95%CI 0.995 to 0.999, P = 0.042).

**Factors associated with 30-day readmission**

Laboratory factors associated with a higher likelihood of readmission in the next 30-days were higher bilirubin (OR 1.004, 95%CI 1.001 to 1.007, P = 0.006), higher ALT (OR 1.002, 95%CI 1.001 to 1.003, P = 0.021), higher GGT (OR 1.001, 95%CI 1.001 to 1.001, P = 0.031), and higher APTT (OR 1.026, 95%CI 1.002 to 1.051, P = 0.035). When examining change in vital sign and laboratory parameters between the 24-hour and 24-to-48-hours periods, a reduction in sodium prior to discharge, an increase in GGT, an increase in PT and a reduction in glucose were all associated with increased likelihood of 30-day readmission.

**Supplementary Table . Cohort characteristics in the 24 hours prior to discharge.**

| **Variable** | **Individuals not readmitted within 30-days (n = 11,780)** | **Individuals readmitted within 30-days (n = 921)** | **P value** |
| --- | --- | --- | --- |
| Age; mean (SD) | 54.6 (20.5) | 55.5 (19.7) | 0.179025319 |
| Female sex; number (%) | 5646 (47.9) | 453 (49.2) | 0.483249368 |
| Socioeconomic percentile; mean (SD) | 59.0 (28.5) | 59.6 (27.7) | 0.572074777 |
| Non-English primary language; number (%) | 1036 (8.8) | 113 (12.2) | 0.000398745 |
| No specified religion; number (%) | 3687 (31.3) | 340 (36.9) | 0.000418 |
| Respiratory rate; mean (SD) | 17.7 (2.3) | 17.7 (1.8) | 0.857385748 |
| Oxygen saturations; mean (SD) | 98.5 (1.6) | 98.5 (1.4) | 0.205197112 |
| For those on supplemental oxygen, O_2_ flow rate | 4.2 (3.9) | 4.4 (7.8) | 0.757204657 |
| Heart rate; mean (SD) | 84.0 (12.9) | 84.5 (13.0) | 0.275054313 |
| Systolic blood pressure; mean (SD) | 137.9 (18.4) | 137.5 (18.2) | 0.546455449 |
| Temperature; mean (SD) | 37.0 (0.4) | 37.1 (0.5) | 0.099940852 |
| Sedation score; mean (SD) | 0.6 (0.6) | 0.6 (0.6) | 0.074471829 |
| Bowel open count in 24-hours; mean (SD) | 0.5 (0.9) | 0.4 (0.9) | 0.218106247 |
| Pain score (/5); mean (SD) | 3.5 (2.9) | 3.4 (2.9) | 0.213161688 |
| Haemoglobin; mean (SD) | 123.0 (18.2) | 122.3 (19.0) | 0.375774811 |
| White cell count; mean (SD) | 8.7 (3.8) | 8.7 (3.5) | 0.799247458 |
| Absolute neutrophil count; mean (SD) | 6.2 (3.2) | 6.3 (3.2) | 0.393002415 |
| Absolute lymphocyte count; mean (SD) | 1.7 (1.3) | 1.6 (0.7) | 0.1931445 |
| Platelet count; mean (SD) | 290.2 (137.9) | 297.1 (151.7) | 0.273765144 |
| Sodium; mean (SD) | 138.4 (3.0) | 138.1 (3.1) | 0.00972854 |
| Creatinine; mean (SD) | 80.0 (53.7) | 77.9 (37.9) | 0.369575053 |
| Urea; mean (SD) | 4.6 (3.0) | 4.6 (2.1) | 0.743023594 |
| Magnesium; mean (SD) | 0.8 (0.1) | 0.8 (0.1) | 0.958894024 |
| ALP; mean (SD) | 118.6 (137.1) | 154.9 (197.3) | 4.57596E-08 |
| AST; mean (SD) | 46.6 (70.2) | 57.4 (87.6) | 0.001355949 |
| ALT; mean (SD) | 56.6 (96.9) | 76.0 (124.2) | 2.69678E-05 |
| GGT; mean (SD) | 126.6 (224.5) | 189.1 (316.8) | 8.20193E-09 |
| Bilirubin; mean (SD) | 13.6 (22.6) | 20.7 (41.7) | 6.37928E-10 |
| Albumin; mean (SD) | 30.8 (4.9) | 30.5 (4.8) | 0.147071902 |
| LDH; mean (SD) | 228.7 (90.0) | 234.0 (80.9) | 0.209807161 |
| Calcium; mean (SD) | 2.2 (0.1) | 2.2 (0.1) | 0.685027055 |
| Glucose; mean (SD) | 6.9 (2.6) | 6.9 (2.8) | 0.671363665 |
| Phosphate; mean (SD) | 1.0 (0.2) | 1.0 (0.2) | 0.711270764 |
| C-reactive protein; mean (SD) | 57.5 (59.0) | 64.6 (65.5) | 0.10199895 |
| APTT; mean (SD) | 31.7 (7.7) | 33.4 (11.5) | 0.08613331 |
| PT; mean (SD) | 16.1 (5.1) | 16.1 (4.7) | 0.979679834 |
| INR; mean (SD) | 1.2 (0.4) | 1.1 (0.3) | 0.90222382 |
